# CyBy^2^: a strongly typed, purely functional framework for chemical data management

**DOI:** 10.1186/s13321-019-0403-2

**Published:** 2019-12-30

**Authors:** Stefan Höck, Rainer Riedl

**Affiliations:** ZHAW Zurich University of Applied Sciences, Einsiedlerstrasse 31, 8820 Wädenswil, Switzerland

**Keywords:** Chemical data management, Lab inventory, Medicinal chemistry, Functional programming, Type driven development, Scala

## Abstract

We present the development of *CyBy*^*2*^, a versatile framework for chemical data management written in purely functional style in Scala, a modern multi-paradigm programming language. Together with the core libraries we provide a fully functional example implementation of a HTTP server together with a single page web client with powerful querying and visualization capabilities, providing essential functionality for people working in the field of organic and medicinal chemistry. The main focus of *CyBy*^*2*^ are the diverse needs of different research groups in the field and therefore the flexibility required from the underlying data model. Techniques for writing type level specifications giving strong guarantees about the correctness of the implementation are described, together with the resulting gain in confidence during refactoring. Finally we talk about the advantages of using a single code base from which the server, the client and the software’s documentation pages are being generated. We conclude with a comparison with existing open source solutions. All code described in this article is published under version 3 of the GNU General Public License and available from GitHub including an example implementation of both backend and frontend together with documentation how to download and compile the software (available at https://github.com/stefan-hoeck/cyby2).

## Introduction

As researchers in the field of drug discovery we have very specific needs when it comes to electronically archiving and visualizing the results produced in our research group. For our daily synthetic work we would like to have an easily accessible lab inventory searchable by molecular (sub)structures with the ability to export selected subsets of the data for the generation of screening libraries or the exchange with external research partners. The inventory should be editable by all researchers, but superusers should be able to review these edits and get comprehensive information about what was changed in the database by whom. To help in the process of drug design, we want to be able to link compounds with activity data from biological assays, and we want to be able to use a powerful but convenient to use querying language together with visualization utilities to analyze these datasets for structure activity relations (SAR) against different targets. The entire SAR data in the database should be accessible to the participating scientists by project affiliation, so that confidentiality of the data is guaranteed, which is of great relevance from an intellectual property rights point of view, especially in industry-related cooperations. In addition, we want to link data objects in our database to files and URLs containing additional information such as spectroscopic data, synthetic procedures, or raw data from bioassays. We also expect the software to prevent us from making common mistakes like entering ill-formed data or duplicate entries into the underlying database. Eventually these requirements led us to implement our own data management tool, going through several stages of refactoring when requirements changed or new functionality was requested [1].

Other groups, however, have different needs. They might want to have a full-fledged electronic lab journal, the ability to not only link spectroscopic data as files but also to be able to query the database for spectroscopic fingerprints or synthetic procedures using certain reactants or having a yield in a certain range. If their needs differ too strongly from what our own data management tool offers, it no longer makes sense for them to use the same piece of software. The authors of the Chemotion ELN have already described the advantages of open source solutions to address these diverse needs [2]. While we agree whole-heartedly, we would like to address an additional issue: Specifications for this kind of software solutions are not static and user requirements change over time. Adhering to these changed requirements poses two major risks: Invalidating the data stored on disk as it no longer matches the new data model and introducing regression errors due to changes made in the code base. Considering that many solutions in cheminformatics consist of thousands of lines of code written in dynamically typed scripting languages like Python (e.g. RDKit [3]), Perl (e.g. Perl bindings in OpenBabel [4]), Ruby (e.g. Chemotion ELN [2]), JavaScript (e.g. ChemDoodle [5]), PHP (e.g. open enventory [6]), or statically but—compared to the languages described below—weakly typed languages like Java (e.g. CDK [7]) or C++ (e.g. OpenBabel [4]), we believe these risks to be real and quite limitating.

One predominant technique used to address the second issue (regression errors) are unit tests: Pieces of code that can be automatically run to verify that the software still behaves correctly. While unit tests play an important role in almost all modern medium to large-scale software projects, they can typically only show the presence of errors but not prove their absence, because in order to do so, a piece of code would have to be tested against all possible values in its domain (the set of possible input values) in all possible environments. While testing a function in different environments is unnecessary if it is referentially transparent (see below), the domains of most functions are far too large to be tested exhaustively in reasonable time. Also, writing thorough unit tests can be cumbersome and time consuming, and as such is easily neglected in favor of adding new features. While enforcing good coding practices like test driven development [8] can help in the writing of more reliable software, we experienced a drastic increase in productivity when turning to writing code in pure, strongly typed functional programming languages thus rendering a large set of unit tests obsolete (see also [9]).

### Pure functional programming

The advantages of pure, strongly typed functional programming languages have already been described in several articles in this journal, and we will only recap the most important points [10, 11]. In functional programming, functions are first class, meaning that functions can be passed as arguments to other functions, can have other functions as their result, can be assigned to variables, and can be stored in data structures. They are the main form of abstraction and code reuse in these languages. Functions taking other functions as parameters or returning them as their results are typically referred to as *higher order functions*.

Pure functional programming languages like Haskell [12] in addition require functions to be *pure*, or *referentially transparent*. An expression is referentially transparent, if it can be replaced with its result after evaluation without changing the behavior of the program whatsoever. As such, referentially transparent functions may not access or mutate global state, make changes to the outside world like writing to or reading from files, interact with peripheral devices or communicate over networks, as all these actions would change a function’s behavior depending on its environment. Pure functions may only operate on their input parameters probably by calling other pure functions, and all values passed to such functions must be *immutable*. While this may seem very restrictive to programmers accustomized to typical imperative languages, pure functions are trivial and safe to compose and easy to reason about. They are per definition safe to be called in a multithreaded setup without the risk of race conditions, deadlocks or other unexpected behavior. Finally, they allow us to come up with mathematical proofs about their correct behavior through equational reasoning [13]. As such they make for highly reusable code components.

While referentially transparent functions can be written in all programming languages, in pure functional programming languages like Haskell or Idris [14] referential transparency is enforced by the type system. Other languages like Scala [15], while being impure by default, are equipped with type systems expressive enough to implement similar effect systems for those who like to keep track about effectful functions at the type level. (One such implementation is provided by the *cats-effect* library [16]).

Algorithms written in purely functional style as well as pure data structures can incur a certain performance cost compared to optimized imperative solutions. For instance, in-place mutation of a field in a complex mutable data object is typically very fast compared to accessing and updating a value in a deeply nested immutable data object. Note, however, that with immutable data the portions of the data structure that are not modified can be shared between the old and new version and therefore need not be copied. For a detailed treatment of purely functional data structures, see [17]. However, even in pure languages like Haskell it is possible to make use of efficient mutable data structures and mutable references if raw performance is required. A function making use of in-place mutation internally is still referentially transparent, as long as the mutable state is securely encapsulated within the function, i.e. is not passed as an argument to the function nor returned as part of the function’s result. Ideally, the safe treatment and proper encapsulation of mutable state can be verified using the language’s type system as is for instance possible in Haskell [18].

### Property based testing

An additional advantage of pure functions is their testability: Being referentially transparent guarantees that these functions always behave the same no matter the environment in which they are called. A common technique to test this kind of function is property based testing: Relations between a function’s arguments and its results are defined and verified against a large amount of randomly generated input [19]. This is especially useful to make sure that type class instances adhere to certain mathematical laws. For instance, the following functions, written in Haskell, verify the laws of reflexivity, symmetry, and transitivity of equivalence relations: 
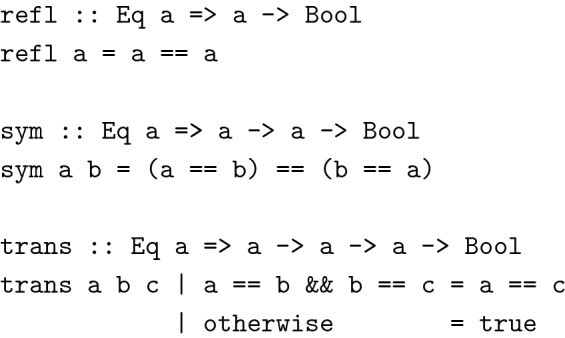


These properties can now be verified for each data type with an instance of type class Eq, by running the functions defined above against a large amount of randomly generated values.

Property based testing leads to great confidence in code correctness as a greater part of a function’s domain is verified in these tests than with manually written unit tests. Typical frameworks like ScalaCheck [20] or QuickCheck [21] make sure to include easily neglected corner cases in the set of randomly generated input values, forcing programmers to take care about typical errors like division by zero or integer overflows, if these cannot already be ruled out at the type level.

### Type driven development

In addition to enforcing or at least encouraging a pure programming style, languages as the ones described above are equipped with powerful, versatile type systems. One technique for writing code in such languages is *type driven development*: Programmers write type level specifications of functions first and with the compiler’s help derive implementations of these functions [22]. Dependently typed languages like Idris [14] can give rise to such detailed type level specifications, that the compiler can in some cases generate code from a function’s type automatically if it can prove that there can exist only one correct, provably terminating implementation. Consider the following trivial example, written in Haskell: 
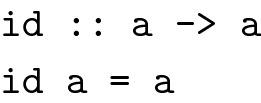


The first line is the function’s type declaration, the second is its actual implementation. The type reads as follows: Given a value of an arbitrary (choosable by the function’s caller) type a, the function returns a value of the same type. It can be shown that this function can have only one terminating, referentially transparent implementation: The function *must* return exactly the value it has been given as input, as it cannot make any assumptions about the value’s type and therefore about its associated operations [23]. While this example might not seem to be very useful, the concept can be extended to more useful type declarations. Consider the following example, relevant to cheminformatics, where molecules often have to go through the right routines of initialization before using them in a given algorithm makes sense. When performing a substructure search, for instance, molecules should probably already have been aromatized and explicit hydrogen atoms should have been added. Toolkits like the CDK usually mention these prerequisites in a function’s documentation, but we consider it to be much more useful, if this information is available at the type-level. The following code snippet (again in Haskell for brevity) describes the concept of using *phantom types* to tag such type-level information to a data type. A phantom type is a type that is never instantiated at run time and serves merely as a type-level marker at compile time. 
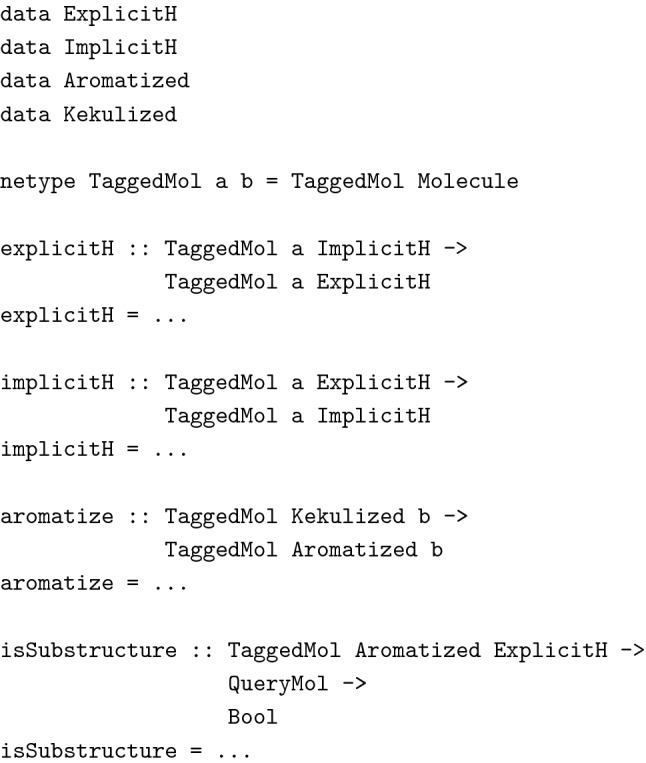


TaggedMol is a wrapper for molecules holding additional type-level information in the form of phantom type parameters a and b. These type parameters are used in the functions described above to keep track of the exact representation used in the molecule. They prevent programmers from aromatizing molecules twice for instance, since aromatize can only be called with a Kekulized molecule, but they prevent us also from performing a sub-structure search on a molecule in the wrong state. Unlike comments in code, tagged types like the ones above are a form of documentation that can never go out of sync with the implementation as it is verified by the type checker whenever the code is being compiled. We hope that this last example shows, how powerful a tool type-driven development is in a programmer’s toolbox.

## Implementation

This section describes the example implementation released together with *CyBy*^*2*^’s source code. Most components can be exchanged depending on preferences as described in section *Results*.

**Fig. 1:**
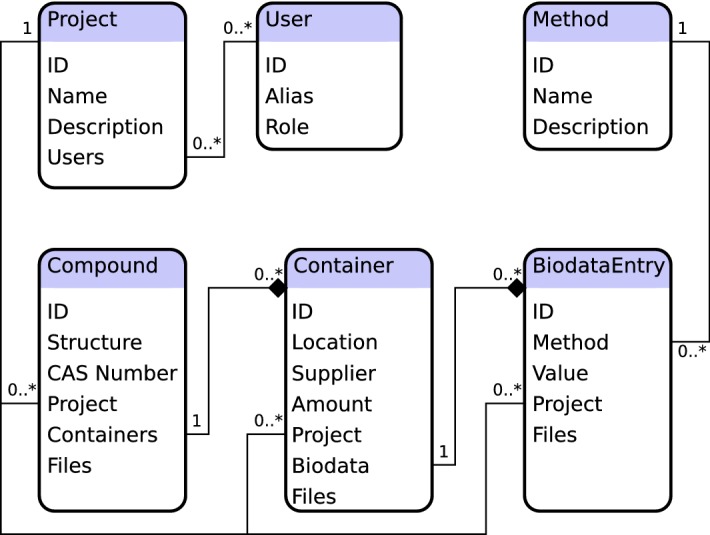
Data Model. This simplified UML diagram shows an excerpt of the data model. Here we see how compounds together with linked files and containers actually form a heterogeneous data tree linked to objects from other “tables” like projects and assays. While it is possible to map these kinds of data graphs to tables in a relational database, we consider tree shaped data formats like JSON or XML to be better suited for this task

Figure 1 shows a simplified UML diagram of the data types used in the example implementation. At the root of the data tree are *Compounds* representing chemical entities typically with a proper chemical structure, name and—if available—CAS number. A compound can be linked to an arbitrary number of physical *Containers* stored at the *Location* given, usually bought from a given *Supplier*. A *BiodataEntry* represents a result from a bioassay represented by the *Method* data type. An arbitrary number of such entries can be linked to a container. Compounds, containers, and biodata entries are linked to *Project*s to guarantee the proper concealment of confidential information. A *User* in *CyBy*^*2*^ has been granted access to a subset of all projects and can view and probably modify only data linked to these projects.

The data model as presented here is flexible and can easily be extended with additional fields or restructured by adding, removing or relinking components. The type checker will reliably guide implementors through this process of refactoring, while a lot of functionality provided by type class instances will be updated automatically (see also the section on Automatic Type Class Derivation). In addition, many of the concepts described in this article are generic and could easily be applied to other fields of science.

With the exception of the CSS rules used in the web frontend, *CyBy*^*2*^ as a whole was written in purely functional style in Scala, a multiparadigm programming language with an expressive type system and strong support for functional programming techniques [24]. Scala was our language of choice since it is compiled to Java bytecode by default, comes with a plethora of useful third-party libraries, and interacting with existing Java libraries is trivial.

We used *sbt* [25] for building the application. The core libraries are split into several modules grouped under a single multi-module sbt project.

The backend consists of a REST server implemented on top of *Http4s* [26], a minimal, purely functional HTTP server based on functional streams (*fs2* [27]). It uses *cats-effect* [16] as its effects system, allowing programmers to wrap calls to impure code in an IO data type making effectful computations visible at the type level. For all chemistry related calculations like substructure and similarity searches the server makes use of the chemistry development kit (CDK [7]). Linked files and user settings are stored in an SQLite database [28], while all other data entries like compounds, containers etc. are stored in a custom JSON format tailormade to allow for the incremental reassembly of the whole dataset. We used the Typelevel Scala Compiler [29] to compile the backend to Java bytecode, as it offers better support for some of the programming techniques used in the implementation.

The frontend consists of a single page web application written also in Scala and compiled to JavaScript using the ScalaJS compiler [30]. For drawing molecules we use ChemDoodleWeb [5]. With the exception of *scalajs-dom* [31], a statically-typed DOM API, the web frontend has no other dependencies on external JavaScript libraries. The interactive behavior of the user interface was implemented using an adaption of monadic streaming functions [32] a generalized functional reactive programming framework originally written in Haskell. The resulting code is available as a module of *CyBy*^*2*^.

Finally, *CyBy*^*2*^ comes with detailed HTML documentation describing its functionality. Documentation is generated by a Scala program having access to the code base of both client and server. As such, the code generating the documentation is strongly typed and reuses the same HTML elements as the web client. This guarantees that examples in the documentation stay in sync with changes made to the core application.

## Results

*CyBy*^*2*^ offers a highly customizable framework for writing chemical data management systems. It comes with powerful building blocks to write reactive user interfaces where users can conveniently analyze datasets in different views, define versatile combined queries including (sub)structure and similarity searches, and quickly add or modify data objects like compounds, linked files, or containers. Selected datasets can be exported to several formats, including .sdf, a standard chemical file format, and .odt readable by spreadsheet applications. In the example implementation, all data objects are linked to a project and users cannot view pieces of information, unless they have been granted access to the corresponding project. With the exception of raw file data and user settings, which are stored in a lightweight SQLite database, changes made to the data are stored incrementally in JSON format and the dataset is reassembled from these changes when the server is started. Administrators therefore have access to the complete editing history of a piece of information, allowing them to easily monitor and review changes made to the data.

### Frontend

Users of *CyBy*^*2*^ interact with the server through its frontend, an interactive single page web application.

#### Queries

*CyBy*^*2*^ offers powerful querying capabilities. It comes with a convenient quick search text field useful for running simple searches. Depending on its format, the search string is either interpreted as a set of compound IDs, a CAS number or a regular expression. Regular expressions are matched against all textual fields in a compound’s data tree, filtering compounds, containers and linked files accordingly.

**Fig. 2:**
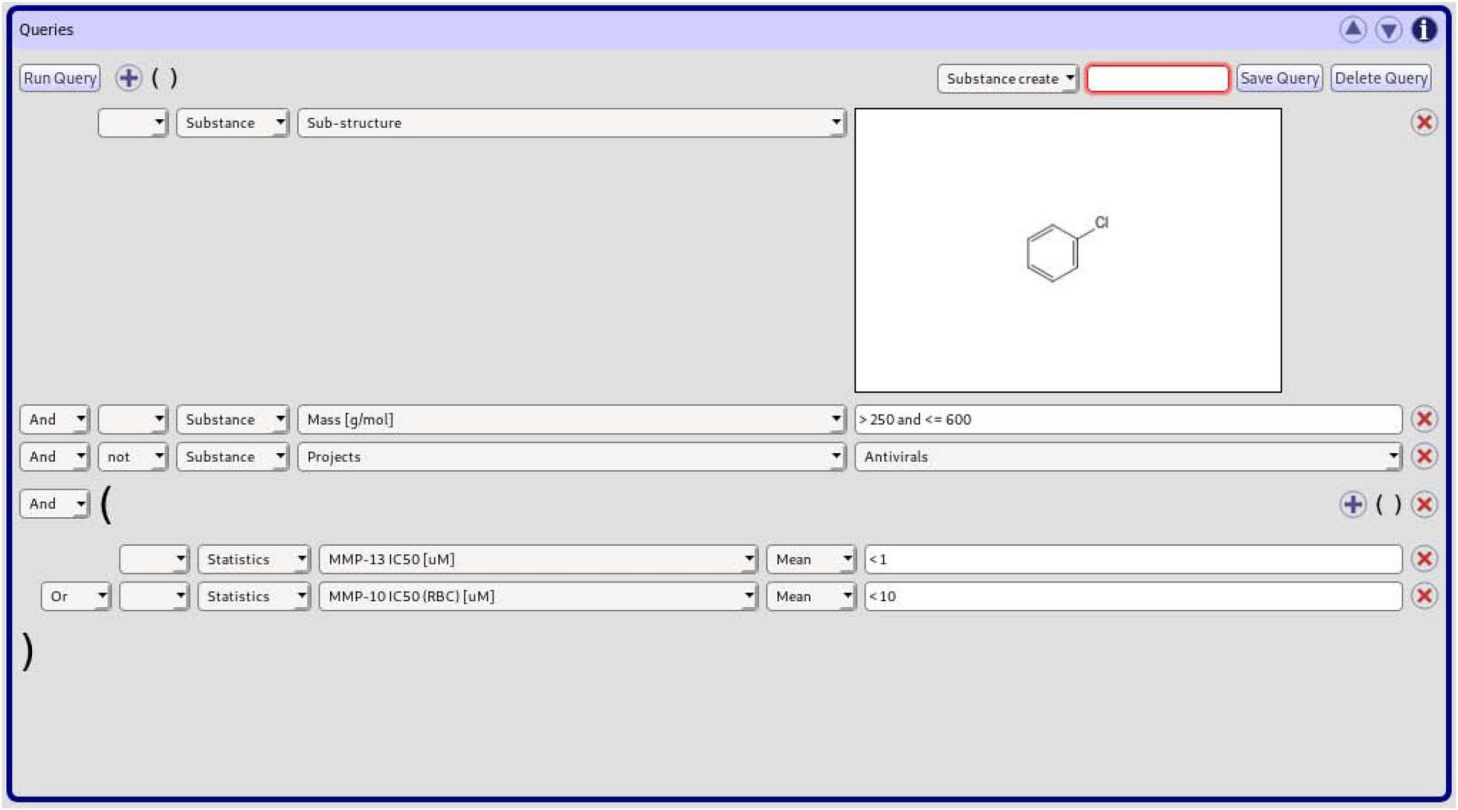
Combined Queries in *CyBy*^*2*^ Each row represents a predicate against a certain piece of information stored in the database. Rows can be grouped in parentheses and combined using logical operators. Often used queries can also be stored to and reloaded from a drop down menu

Advanced users can make use of *CyBy*^*2*^’s capabilities to define combined queries (Fig. 2). Every row represents a predicate tested against one field in the heterogeneous data tree. The type of query changes dynamically with the selected field: Numeric queries allow users to enter a combination of numbers and comparators, textual fields come with a text input together with a dropdown to define how the query should be interpreted. Queries against links to other data objects like suppliers, locations or projects come with a dropdown menu containing all valid options depending on the logged in user. Rows can be combined using logical operators and lists of rows can be grouped in parentheses, leading eventually to a tree of predicates to be sent to and interpreted by the backend. An arbitrary amount of structure based queries like substructure and similarity searches can be included in a combined search. Finally, often used queries can be given names and persisted together with other user settings.

At the backend an interpreter for combined queries consists of a function returning a parser for predicates depending on the field subjected to the query. Fields are just enumeration-like data types closely related to the actual structure of the data types used to represent compounds and containers. The compiler can be made to enforce pattern matches against fields to be exhaustive and thus not a single case to be missed. This concept of defining behavior depending on a selection of fields comes up again, for instance when exporting data or when displaying data in tabular form with a selection of visible rows.

#### Data visualization

**Fig. 3:**
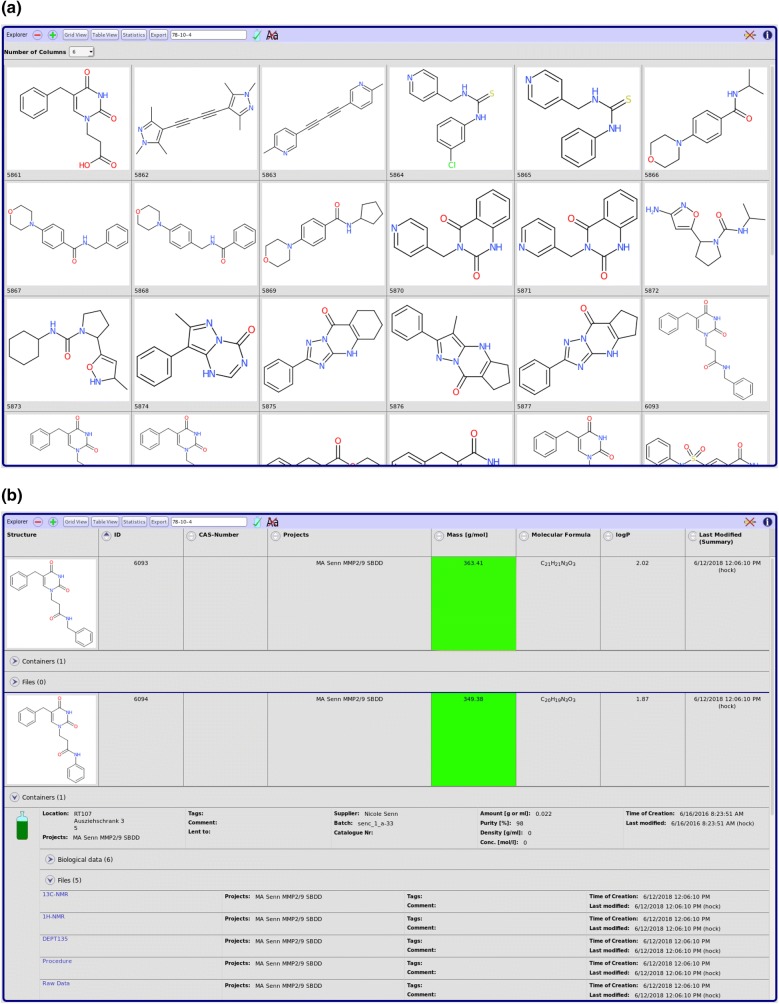

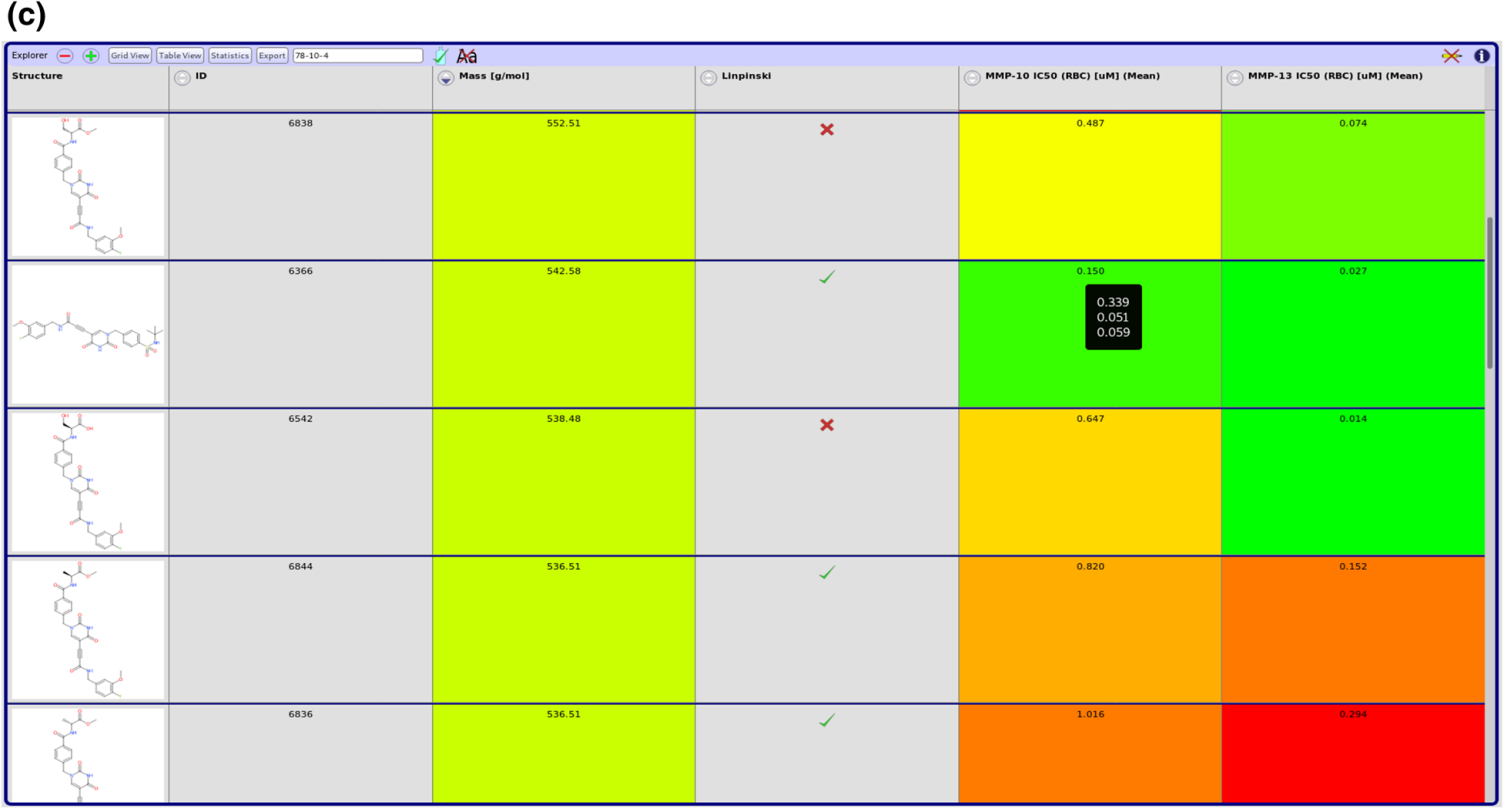
Data visualization. Hit sets from queries can be visualized using different views. For a quick overview and a convenient way to select a subset of the compounds returned, the grid view can be used (**a**). The default view is an expandable tree closely related to the tree shape of the underlying data (**b**). A tabular view is used to analyze structure activity relations (SAR). Background color gradients can be defined for numeric columns to help with the visualization of data (**c**)

Hitsets from queries can be displayed in several views (Fig. 3). The default tabular view actually consists of expandable nodes reflecting the tree structure of the underlying data model. The selection and order of displayed columns is customizable and the settings persisted together with other user settings. For a quick overview a grid view displaying just the structures of compounds is available. Subsets of compounds can be conveniently selected for instance to export only parts of a hitset. For analyzing structure activity relations another tabular view grouping entries by batch is available. Here, additional columns with statistics of biological activities can be displayed. For numeric columns, color gradients can be defined to help with the visual interpretation of the data.

#### Lazy loading

Since hitsets from queries can be quite large, consisting of thousands of compounds, in order to not slow down the UI only small packages of results are loaded at a time. In order to view additional results, users can just scroll down in the different views. Upon getting close to the bottom, new data is automatically requested from the server.

#### User roles and data editing

Users in *CyBy*^*2*^ can be assigned different roles ranging from guest to administrator accounts. Most users are allowed to make changes to the data. Editing data is turned off by default in order to prevent users from inadvertently making changes when interacting with the user interface. It can be enabled by clicking on a master button in the explorer. All changes are persisted together with a timestamp and user ID. This allows superusers and administrators to peer review changes made to the data and get in touch with users who submitted data of insufficient quality. Several combined query options are available to facilitate this kind of administrative task.

#### Exporting data

**Fig. 4:**
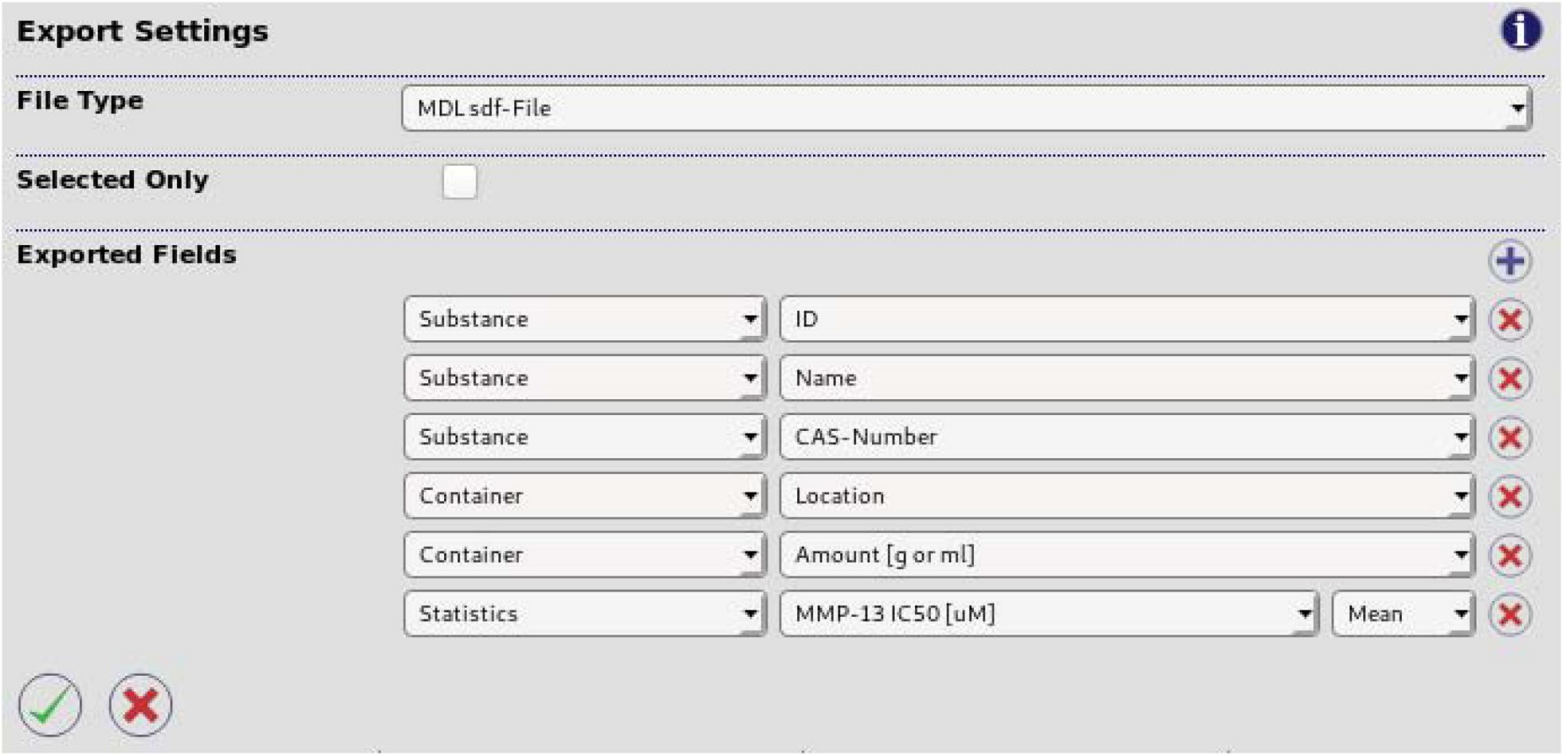
Exporting data. *CyBy*^*2*^ gives users detailed control over what fields to export in what order. Fields can be selected from the drop down menus and new columns can be added by click the ‘plus’ icon. Several different file formats are available for exporting

Results from the latest query can be exported to several file formats (Fig. 4). Users can freely add to or remove from the list of exported fields. This selection not only determines the columns in tabular file formats but also the number of rows. If only fields of compounds are selected, there will be one row per compound. However, if fields of containers are included, there will be one row per container. *CyBy*^*2*^ supports exploring to .sdf, .odt (to be read by spreadsheet applications) and .csv (tab delimited).

### Backend

The server is responsible for providing the core functionality of *CyBy*^*2*^. This includes loading, persisting, validating, and updating of data, querying and sorting of data, exporting of data to different formats as well as user management, authentication and authorization. As such, the server plays a critical role for *CyBy*^*2*^ to operate correctly. Resorting to a purely functional, strongly typed programming style allowed us to be confident in the correct behavior of the server even in the face of aggressive code refactorings.

### Chemistry toolkit

The server uses the CDK for all tasks related to computing properties of compounds and performing structure-based queries like substructure or similarity searches. Since strictly speaking, all code in the CDK is unsafe (referentially opaque), we provide safe wrappers for the core functionality needed by *CyBy*^*2*^. In order to make CDK’s functionality available from within pure code, we do not wrap mere calculations like—for instance—the ones for getting the mass of a molecule or performing substructure searches in the IO monad, but in a wrapper type guaranteeing the confinement of mutable structures to the implementation of pure functions. This technique is also used in Haskell for instance to use mutable arrays when implementing performance critical, referentially transparent functions [18]. In addition, return types of our wrapper functions always reflect the possibility of failure for these calculations. This was necessary, since in the CDK fields of data objects are often initialized to null (probably for performance reasons) and NullPointerExceptions occurred frequently when working with objects which have not gone through the necessary initialization routines. Wrapping these calls in the Either monad allows us to provide additional information about the input parameters giving rise to an exception and programmers are forced by the type system to eventually break out of Either thereby handling all exceptions that occurred during a calculation.

### Persistence layer

The persistence layer of an application in its most basic form reads and writes data from and to disk. Typically, this is done using some kind of relational database such as PostgreSQL [33]. Queries are then either run directly against the data on disk, or all data is first loaded into memory and managed by the server application. The latter typically is faster but works only up to medium sized datasets fitting still in the server’s memory.

While *CyBy*^*2*^ can easily be linked to any persistence framework such as doobie [34] through mere function composition, we preferred the latter approach whenever possible due to the increase in type safety and possibly performance. Instead of laying out data as tables in a relational database, we stored data incrementally in JSON format. This had several advantages:

- Most importantly,our in-memory model was much more of a natural fit: In contrast to the flat tables used in relational databases, data objects in applications such as *CyBy*^*2*^ are better modelled as heterogeneous trees (Fig. 1). While assembling heterogeneous data trees from relational databases is of course possible, the necessary SQL queries can be cumbersome to write and slow in performance. This motivated the approach of NoSQL systems for storing data in non-relational formats. The JSON format offers a lightweight NoSQL solution: JSON objects are (mostly) untyped heterogeneous trees. As such they are a natural fit for storing our data. In addition, encoders and decoders from and to JSON could be conveniently derived automatically for regular algebraic data types, using the *circe* library [35].
- A custom persistence model allowed us to store *changes* to the data instead of just overwriting existing rows in databases tables. Upon starting the server, the whole dataset is incrementally reconstructed from its history. As such, we always had access to the complete history of the data and could make this history available to administrators for reviewing changes made by users.
- We often had to make adjustments to the data model such as when adding new fields or supporting new data types due to evolving requirements of end users. With an in-memory model based on a JSON encoding, we found it to be trivial to allow for such changes: New fields were typically optional (wrapped in an Option[A], a functional programmer’s typesafe alternative of null). In case they were mandatory, we could provide default values probably calculated from other fields. All this could easily and safely be handled by the server. At no point did we need to touch or modify the data stored on disk. Fields missing from a JSON tree already stored on disk were automatically loaded as None forcing us at the type level to provide default values if necessary.

This approach worked very well for datasets fitting into the server’s memory as a whole. However, care had to be taken to make sure that calls to mutate the data (both in memory and on disk) are properly synchronized and occur strictly in sequential order while mere queries can be parallelized freely. The implementation uses an MVar provided by the *cats-effect* library [16]. This is a thread-safe mutable variable, that can either contain a value or be empty and can act as a binary semaphore to make sure only one thread at a time can access and modify mutable state and write to disk.

Groups with larger datasets might consider a hybrid approach: As chemical structures together with their fingerprints required in substructure and similarity searches typically make up the bulk of a chemical database, this information can still be stored in a relational database and these kinds of queries run using a chemical database cartridge such as RDKit [3] or Sachem [36], while additional structured data is still stored as a data tree. (A database cartridge is a way to enhance an existing database implementation with business logic from other domains. RDKit, for instance, provides a cartridge to enhance a PostgreSQL database with capabilities for substructure and similarity searches in molecular graphs stored within the database). While such a hybrid system has not yet been implemented in *CyBy*^*2*^, it should be straight forward to do so without significant changes to the remainder of an already existing code base, once datasets get large enough.

There is one exception to our JSON-based approach: We stored linked files and user settings in a local SQLite database without keeping track of their update history. Files can occupy large amounts of space and it makes no sense loading them into memory as a whole. User settings on the other hand change with almost every client request. As such it would take up too much space and we would gain very little if we stored these changes incrementally.

### Data model

The main advantage of writing both the backend and frontend of a web application in the same strongly typed programming language is the large amounts of code the two parts can share. This is especially useful when it comes to sharing the data model, since correct protocols for encoding and decoding data come for free this way. In this part we are going to describe some of the techniques used to write detailed type level specifications and to make use of those specifications in order to derive all kinds of behaviors generically.

#### Flexible data types

The code samples below have been considerably simplified compared to the data definitions in the actual source code and some of the classes and functions are used solely to demonstrate how our data model evolved. These are not part of the source code. However, where package names are given explicitly, class names are the same as in the source and should therefore be easy to locate for interested readers.

While sharing the data model between frontend and backend immediately sounded reasonable, it was at first not clear how to do this properly because when client and server communicate with each other, they necessarily have to represent some data types differently, be it for reasons of performance, confidentiality or simply lack of information. For instance, consider the following stripped down example of a chemical compound: 
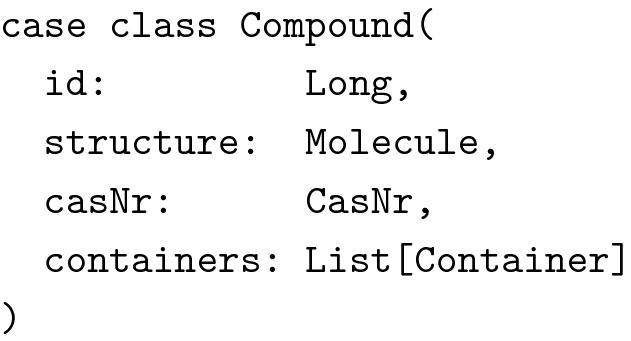


Since we used the CDK at the server (running on the Java Virtual Machine) for handling chemical structures, it was not possible nor desirable to use the same representation at the client (JavaScript running in the browser). Therefore, while the server of course had to know about molecules, the client did not and even could not. The client only required a vector graphics representation to display compounds most of the time. So we actually needed two data types for molecules: One for the data stored in memory at the server, one for the data to be sent to and displayed by the client. 
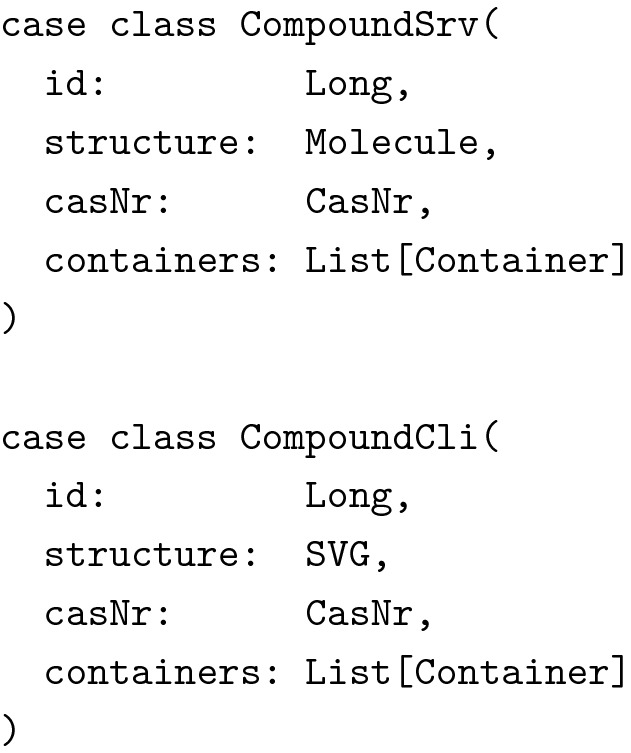


Note, how the two data types are not related through a common superclass. We wanted to be precise about the types and not mix them up in any way. We also wanted to use the same data type to send requests from the client to the server to create new compounds, as well as for updating existing compounds. This introduced several new problems. First of all, the structure’s type was again wrong: We could not use vector graphics to describe molecular graphs and CDK’s Molecule data type was not available at the client. In addition, we did not want the client to dictate the server what ID to use for new compounds. Also, we wanted to separate the creation of new compounds from the creation of new containers. Reflecting this in the types, we arrived at the following additional data type: 
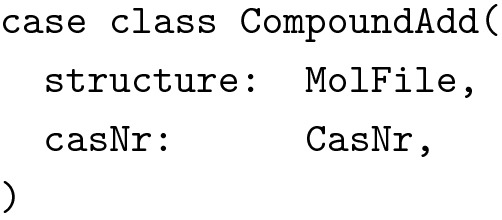


Given the next available compound ID and a function to read molecules from mol files, it was now trivial to implement a utility function mkSrv for creating compounds from CompoundAdd objects. 
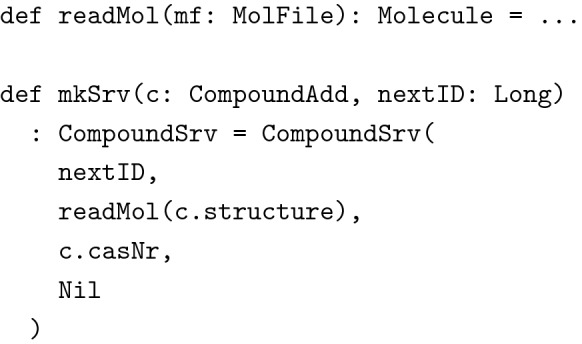


Note how we eliminated several possibilities for erroneous behavior. The types guarantee, that the structure is a well formed MolFile and that the compound’s CAS number adheres to the desired invariants. But the types also guarantee, that the server is responsible for creating new compound IDs and that no containers are added for instance by sending a forged HTTP request to the server. (Note: The types in this example have been simplified for clarity’s sake. In the actual implementation we used a wrapper type for hiding the mutable internals of Molecules and the result type of readMol had to reflect the possibility of failure when reading the molecule from a text representation.)

But this data type was not well suited for modifying compounds, as users usually do not want to modify all fields simultaneously. Of course we could just copy the other fields and send them back to the server, but this would mean that every change made for instance to the name of a compound, would also lead to the storing of the compound’s structure, unnecessarily increasing the size of the database. We therefore wrote another data type, where all fields were optional. 
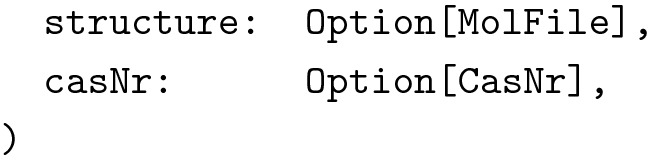


This lead to a collection of data types around the concept of a compound, each with clear properties documented at the type level. Interactions between these data types, for instance when creating new compounds or when sending compounds to the client, were trivial to implement correctly since most mistakes would immediately lead to type errors. While we thus had greatly improved the type level specification of our data model, we also had drastically increased the amount of code, considering that we had to provide implementations of JSON encoders and decoders together with other type class instances for each of these classes and that the real versions could consist of dozens of fields.

Using a polymorphic data type (higher-kinded in one type parameter) together with Scala’s ability to define type aliases solved this issue quite nicely. The actual polymorphic data type was defined in the data module shared by client and server. 
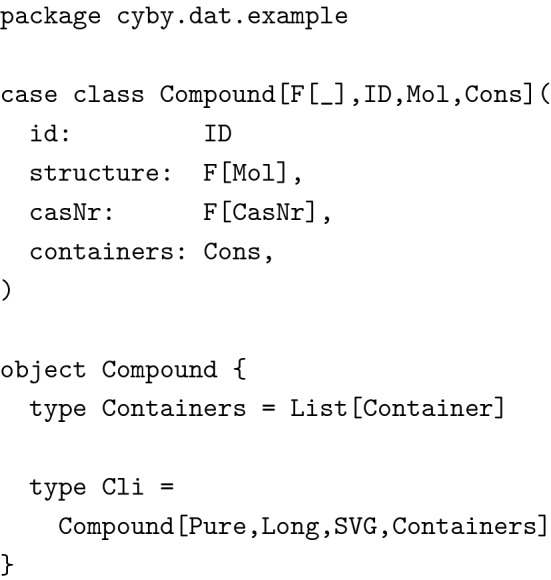


Type aliases only used at the server were defined within a wrapper object in the server module. 
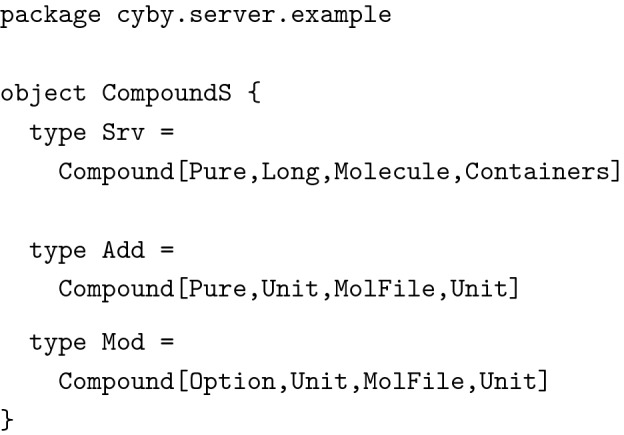


Data type Compound was now polymorphic in most fields (with the exception of casNr), leading to great flexibility about what types of data were actually bundled with a compound while keeping the name of fields consistent. The most interesting part is the higher kinded parameter F[_]. It describes the *context* in which values appear. Typically, it was set either to Pure, meaning that all values had to be present or to Option, meaning that values were optional, which reflected our needs for updating data. Fields not used by some representations were set to Unit, a type inhabited by just a single value. These type declarations lead to the same behavior and guarantees as the different class declarations described above but without the code duplication. We were able to define additional type aliases for instance for compounds after user authorization and input validation, allowing us to enforce important invariants about our code at the type level. The technique described here was used excessively in the example implementation.

#### Confidence at the type level

We want to give one other example, again slightly simplified, how we made use of types to enforce certain invariants in our code. We wanted to prove, at the type level, that access to data objects like compounds had been properly verified before sending them to clients. This was critical, since we did not want to leak information to unauthorized users. For this we defined a simple polymorphic wrapper type with a private constructor: 
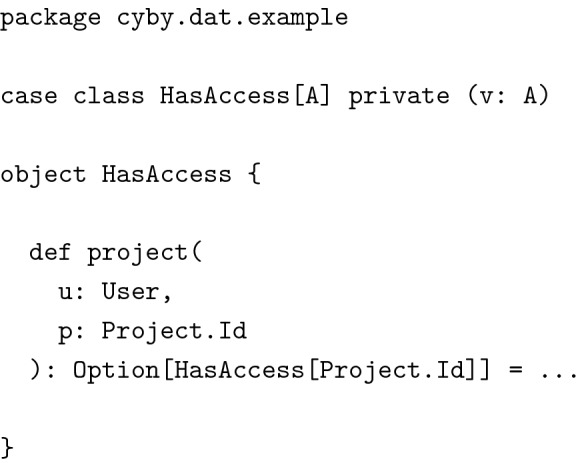


(Note: In the source of the example application, function project is available through a helper class AuthEnv, which we have omitted here for increased readability). We used projects to grant access to compounds and we tagged project IDs with HasAccess before sending data to clients. 
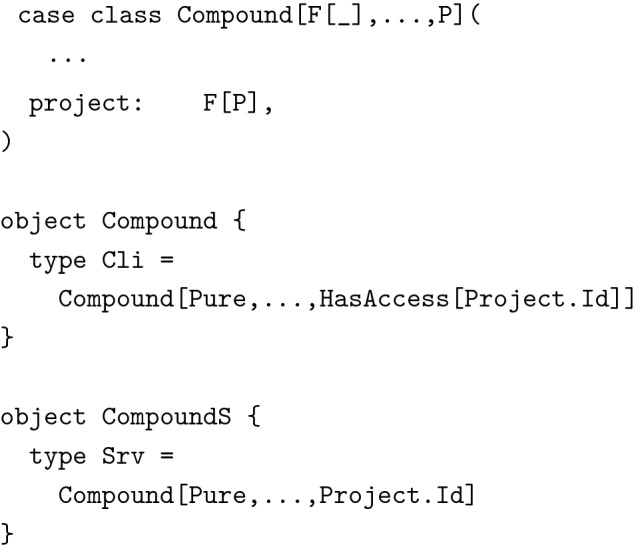


The *only* place from where we could get an instance of HasAccess[Project.Id] was the corresponding function in HasAccess’s companion object. This proves, at the type level, that whenever we sent a response of type Compound.Cli to the client, access had been verified. Of course we still had to check via unit tests, that the implementation of HasAccess.project was correct but this was only a small piece of code, easily testable using property based testing. Techniques like these allowed us to drastically reduce the surface area of functions that actually required testing. The rest of the application could be safely glued together with the help of the type checker.

This last example shows the amount of confidence we could get from a minimal amount of code and descriptive, flexible types. We used similar techniques to prove that data had been properly validated before being stored, and delicate information like hashed passwords were not accidentally being sent to clients.

#### Automatic type class derivation

Algebraic data types like the ones described above are typically made up of two core building blocks: Sum and product types. For these data types it is possible to automatically derive an isomorphic, canonical representation together with conversion functions to and from this canonical form [37]. If for a given type class (for instance JSON encoders and decoders) we can write implementations for the canonical building blocks, we can also have implementations for the corresponding algebraic data types. This generic type class derivation is a powerful concept and helps to drastically reduce the amount of rather uninteresting code necessary to implement type classes. Unlike Java libraries like *gson* [38], this happens at compile time without the need to resort to runtime reflection resulting in robust, type safe code. Two Scala libraries provide the necessary functionality: *shapeless* [39, 40] for automatically generating generic representations of data types, and *circe* [35] to derive JSON encoders and decoders for these generic representations.

This approach was not only used when deriving JSON encoders and decoders. We used it also in the UI to automatically derive the generation of forms for creating new data objects and at the server to merge updates into the data tree and aggregating data objects with information from weakly linked data types before sending them to the client. Once again this enhanced the flexibility of our data model: After adding new fields to existing data types, or after changing the types of existing fields, recompiling the application would either result in compilation errors if type classes could no longer be derived automatically or type class instances were automatically adjusted to the new data representations behaving correctly without further ado. In case of compilation errors it was obvious most of the time how to satisfy the compiler by manually providing additional type class instances for every component of an algebraic data type.

#### Exchanging parts of *CyBy*^*2*^

We think we made some reasonable choices when implementing our example application, but users of our library might want to exchange some parts, for instance to use an existing relational database. This is of course possible. The beautiful thing about strongly typed functional programming is that the main building blocks are just pure, well typed functions. Functions can easily be exchanged for other functions of the same type using the help of the type checker to glue components together. For instance, below is the type of an HTTP request to run a query against the data stored in the server’s memory: 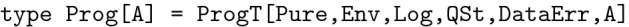


While this may look intimidating, it is actually a quite accurate specification of what we can expect from a value of this type. It is just an alias for a function of the following type, wrapped up for better composability (since types in Scala and other strongly typed functional languages can get quite verbose, type aliases are often used to make code more readable): 
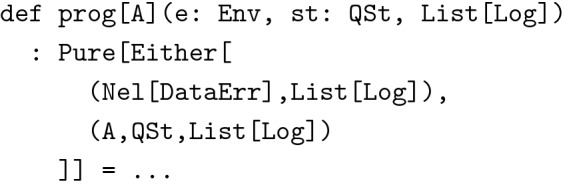


So, prog takes an immutable environment of type Env (a purely functional form of dependency injection), an immutable state of type QSt, and a list of logs, and either returns a non-empty list of DataErrs plus as list of logs or a result of type A together with an updated QSt and a list of logs. The wrapper type Pure describes the side effects this program can have when finally being executed. In this case this means no side effects whatsoever. To be a bit more precise: Env holds all information about the HTTP request together with the data tree currently stored in memory and information about the already authenticated user who made the request. We need this to filter results according to the projects the user has access to. QSt is data that can change after a query has been processed. It could for instance be used to cache the results of queries in order to reduce response times. DataErr is an algebraic data type representing all the ways, in which a request at the server can fail. If such an error occurs, it is both written to the log and sent back to the client, which translates it into human readable form and displays a message in the user interface.

We want to emphasize once again that all values passed to prog are immutable. As such it is impossible for function prog to change the global state of the server application. Even in the case of QSt the state returned by prog is a copy of the state object passed to prog as an argument probably with some fields updated. It is the responsibility of the *caller* of prog what to do with the updated state. This gives us a clear separation of concerns visible at the type level. However, function types like the one of prog can be cumbersome to compose. That’s why they are usually hidden behind polymorphic wrapper types called *monad transformer stacks*, for which one can write instances of type class Monad, thus greatly increasing their composability without compromising type safety [41].

If we wanted to change the way queries were handled, for instance by switching to a relational data base, we would first adjust prog’s type accordingly: We would probably still be using the same mechanisms for caching (if any), but Env would no longer hold an in memory copy of the data. On the other hand it would contain information about the database connection to be used. The effect type Pure would have to change in order to reflect that we now need to access an external database. The type checker would then guide us to make sure that all types match up again once we glued this new component together with the rest of the application. This is the essence of type driven development: Specify types first and let the type checker guide you towards a correct implementation.

### *CyBy*^*2*^ in the context of cheminformatics

Having described above in detail the advantages we experienced from the design choices made in *CyBy*^*2*^, this section will talk about some of the requirements necessary to get started with using *CyBy*^*2*^ as a framework to write custom data management applications.

As a framework written in purely functional style in Scala, *CyBy*^*2*^ will require certain efforts from scientists and programmers used to write code in imperative, object oriented languages. Scala has the advantage of having access to a plethora of Java libraries such as the CDK already existing in the fields of cheminformatics and science in general, and calling Java code from within Scala is trivial. In addition, native code from libraries written in C or C++ can be called from with Scala as well as Java through the Java Native Interface. As such, adopters can go ahead and freely use a large amount of libraries available in Java and other languages together with *CyBy*^*2*^’s Scala code base. However, typical design patterns used in object oriented languages such as those proclaimed by the famous *Gang of Four* [42] have little to no meaning in the realm of pure functional programming, while abstractions from category theory like *functor*, *monoid* or *monad* being used in many places in *CyBy*^*2*^’s source code are foreign to programmers new to strongly typed functional programming. Adopters of *CyBy*^*2*^ will therefore be required to get a firm grasp on these algebraic concepts and we would like to give some recommendations in terms of literature we deem to be easily accessible for people interested and new to this topic. *Functional Programming in Scala* [24] gives a thorough introduction to writing pure, precisely typed functions and how to make good use of the abstractions mentioned above. Being written in Scala is an additional advantage for people wanting to get started with using *CyBy*^*2*^ as the foundation of their own data management tool. Many more resources about pure functional programming exist for the Haskell programming language (see for instance [43, 44]), and indeed we think Haskell—being pure by default—to be a very good choice for learning functional programming from the very beginning.

## Conclusion

*CyBy*^*2*^, a flexible open source framework for writing pure, strongly typed chemical and biological data management applications was described. *CyBy*^*2*^ comes with a fully operational example implementation of an HTTP server and a single page web client, capable of running complex combined queries including substructure and similarity search, lazy loading of large datasets, different views for visualizing and analyzing data, and support for exporting selected pieces of information to several common file formats. Considering its capabilities, *CyBy*^*2*^’s code base is very lean, consisting of only about 10’000 lines of Scala code.

To the best of our knowledge, *CyBy*^*2*^ is the first example of a chemical and biological data management tool written in purely functional style. As such it can also be seen as a resource of coding practices in functional programming in a real world application. It was designed with the diverse and evolving needs of research groups, governmental organizations and industry in mind, requirements we have evaluated both in-house as well as together with collaboration partners from academia and industry. These needs include the ability to link diverse information to chemical structures allowing users to easily access this information through an intuitive to use, well documented web interface and providing powerful and flexible capabilities for querying and exporting the data. At the same time the underlying data model should be flexible enough to allow for the smooth evolution of the data handled by the application, as requirements regarding the information available change regularly. Since adjustments to the data model pose several risks as has been outlined in the introduction of this article, we found the reliability provided by a strongly typed data model to be highly valuable with regards to the constant refactorings required for evolving the software. During refactoring, the power of a modern expressive type system helped us when verifying that components interacted correctly and important invariants were being upheld, but also with the automatic derivation of type class instances to provide overloaded functionality. We use *CyBy*^*2*^ intensively as the central data processing tool in our research group. In recent years, it has proven its value for the design, synthesis and analysis of our drug molecules in complex medicinal chemistry projects [45–50].

Since server, client, and documentation were compiled from the same code base, we could reuse a considerable amount of code between these three parts of the application, thus reducing the possibility of bugs when defining the protocol for exchanging data and making sure that the different parts of the application stayed in sync even in the face of serious refactorings.

We plan to add additional functionality to the project found in other lab notbook and inventory applications such as Chemotion ELN [2] or open enventory [6], giving these features a proper description at the type level to facilitate their safe incorporation into custom instances of *CyBy*^*2*^. Most importantly, we plan to include functionality to query major suppliers’ webpages by CAS number to retrieve chemical structures and safety data. Also missing are ways to view, analyze and query spectroscopic data uploaded as raw data files. We are also working on a layer of more accurately typed wrappers for functionality provided by the CDK in order to facilitate writing code that works correctly without throwing exceptions once it compiles, as we are used to as functional programmers.

An alternative to static type systems for proving code correctness is the formal verification of software using satisfiability modulo theories (SMT) solvers. The *stainless* library allows programmers to define pre- and postrequisites of functions for a subset of the Scala language, which are then formally verified by an external SMT solver [51, 52]. Since we made use of parts of the language not yet covered by *stainless* (for instance higher-kinded types), these techniques have not yet found their way into our code base, but we will observe with interest the progress being made in this regard.

## Data Availability

Availability and requirements: Project name: CyBy^2^. Project home page: https://github.com/stefan-hoeck/cyby2. Git tag of version used in this article: v0.3. Operating system: Platform independent. Programming language: Scala. Other requirements: HTTP server like Apache HTTP Server 2.4. License: GNU GPL. Any restrictions to use by non-academics: None (see GNU GPL).
